# Correlation between LncRNA‐LINC00659 and clinical prognosis in gastric cancer and study on its biological mechanism

**DOI:** 10.1111/jcmm.16069

**Published:** 2020-11-03

**Authors:** Yongjia Sheng, Chenyang Han, Yi Yang, Jin Wang, Yanling Gu, Wenyan Li, Li Guo

**Affiliations:** ^1^ Department of Pharmacy The Second Affiliated Hospital of Jiaxing University Jiaxing China; ^2^ Department of Center Laboratory The Second Affiliated Hospital of Jiaxing University Jiaxing China

**Keywords:** cell cycle, gastric cancer, invasion, lncRNA‐LINC00659, SUZ12

## Abstract

Non‐coding RNAs play important roles in tumorigenesis and tumour progression. In previous screening, lncRNA‐LINC00659 (LINC00659) is highly expressed in gastric cancer; however, its role in gastric cancer has not been illustrated yet. In this study, the expression of LINC00659 was detected in cancer tissues and paracancerous tissues of patients with gastric cancer. As a result, LINC00659 expression was increased in gastric cancer tissues, which was closely associated with tumour stage and lymph node metastasis, but was not correlated with age, gender and tissue differentiation. Survival curve analysis showed that patients with low expression of LINC00659 harboured higher overall survival. In vitro, the level of LINC00659 was increased in gastric cancer cells. Afterwards, the expression of LINC00659 was down‐regulated in SGC‐7901 and BGC‐823 cells by plasmid‐mediated si‐LINC00659 transfection. Consequently, the cell invasion ability was weakened, the cell cycle was inhibited, and cell viability was also suppressed. Luciferase reporter gene assay and RNA pull‐down assay showed that LINC00659 could bind to the transcription factor SUZ12, indicating that SUZ12 was a regulatory gene of LINC00659. The overexpression of SUZ12 could resist the roles of si‐LINC00659. In this study, we found that LINC00659 was highly expressed in gastric cancer, which might be related to the regulation of cell proliferation and promotion of cell invasion. Transcription factor, SUZ12, was a regulator of LINC00659. Additionally, LINC00659 could regulate cell cycle and invasion of gastric cancer by promoting the expression of SUZ12.

## 1 BACKGROUND

Long non‐coding RNAs (LncRNAs) are a type of nucleotides with over 200 nt in length, able to regulate gene expression. LncRNA can be divided into sense LncRNA, antisense LncRNA and bidirectional LncRNA. LncRNAs can regulate gene expression at the post‐transcriptional level, playing an important role in protein synthesis, maturation and transport of RNA.^1^, ^2^ Gastric cancer is a malignant tumour with high morbidity and mortality. The degree of tumour invasion, lymph node metastasis and treatment is important factors affecting the prognosis of patients with gastric cancer, all of which are involved in the proliferation and invasion of tumour cells.^3^, ^4^ Present reports indicate that a large number of abnormally expressed LncRNAs are present in gastric cancer, and the majority of LncRNAs are associated with metastasis, invasion and poor prognosis of gastric cancer. For example, HOTAIR,^5^ a non‐coding RNA of the antisense strand of the HOX transcript, can regulate chromatin status and cell proliferation. H19, a gene product playing a role in imprinting, promotes gastric cancer metastasis by regulating c‐Myc.^6^ Due to the close relationship between LncRNA and gastric cancer, LncRANs, abnormally expressed in tumour cells, can be used as tumour markers to diagnose gastric cancer. Previous studies have found that HOTAIR can be used as a critical marker for the diagnosis and prognosis of breast cancer.^7^ SUMO1P3, a small ubiquitin‐related LncRNA, is also closely associated with the clinicopathological characteristics of patients with gastric cancer. The expression of SUMO1P3 is significantly higher in gastric cancer than that of paracarcinoma tissue; additionally, SUMO1P3 can also be used as a marker to distinguish benign gastric ulcer, gastric polyp and gastric cancer.^8^ In our previous screening, LINC00659 is highly expressed in gastric cancer; however, its role in gastric cancer and its possible correlation with pathological parameters have not been revealed. Therefore, in this study, we mainly investigate the correlation between LINC00659 and clinical and prognostic factors in patients with gastric cancer through clinical samples, and to reveal the mechanism of LINC00659 in metastasis and invasion in gastric cancer.

## 2 MATERIALS AND METHODS

### 2.1 Collection of clinical cases and samples

A total of 80 paraffin samples were collected between 2013 and 2015, who underwent surgery for gastric cancer and were pathologically confirmed as gastric cancer. The inclusion criteria were as follows: (a) The tumour tissues and paracarcinoma tissues of patients were pathologically confirmed as adenocarcinoma, and other diseases, such as metastatic cancer and lymphoma were excluded. (b) Adjuvant radiochemotherapy was not performed on any patients. (c) Patients were not combined with other types of malignancy. (d) The study was approved by the Ethics Committee, and patients or their families signed informed consent. The clinical data included age, gender, tumour size, differentiation degree, lymph node metastasis and TNM stage according to the sixth edition of the International Union Against Cancer (UICC) staging criteria. Surgical samples were stored in RNAlater for further use.

### 2.2 Extraction of total RNA from tissues and detection of relative expression of LINC00695

A total of 50 mg cancer tissue/adjacent tissue was cut into piece by sterile surgical scissors, followed by addition of 600 μL of Trizol reagent (Invitrogen) to the centrifuge tube. The tissue was ground, homogenized for 2‐3 minutes until no presence of solid mass. The homogenized sample was then transferred to a 1.5 mL centrifuge tube, followed by addition of 600 μL of Trizol reagent and subsequent incubation for 5 minutes at room temperature to separate the nucleic acid and the protein. Proper amount chloroform was added (according to the proportion that 0.2 mL of chloroform/1 mL of Trizol reagent), shaken for 15 seconds, incubated at room temperature for 2‐3 minutes and centrifuged at 3000 *g* for 20 minutes. Centrifuged sample was divided into three layers, the upper layer contained RNA, which was transferred to RNase‐free centrifuge tube, followed by addition of an equal volume of isopropanol to precipitate RNA and incubation at room temperature for 10 minutes. After centrifuging at 3000 *g* for 15 minutes and drying, 50 μL of RNA‐free water was added dissolve RNA, followed by purity detection. In brief, UV spectrophotometer was used to measure the absorbance at 260 and 280 nm, and the ratio of OD260/OD280 was calculated. OD260/OD280 between 1.8 and 2.0 indicated qualified purity of RNA sample.

Reverse transcription of RNA: After preparing the reverse transcription system, the reverse transcription reaction was performed at 55°C for 30 minutes, and the reverse transcriptase was inactivated at 85°C for 5 minutes. Finally, PCR tube was placed on ice for 5 minutes to terminate the reaction, and the cDNA product was stored at −20°C for further use.

Reaction conditions of RT‐qPCR: Primer sequence of LINC00659: Forward 5'‐ACCCCTGAAGGACCATATCCA‐3'; Reverse 5'‐GGCTCGGCTGTGTCTCAAG‐3', and GAPDH (internal reference): Forward 5'‐TGCACCACCAACTGCTTAGC‐3'; Reverse 5'‐GGCATGGACTGTGGTCATGAG‐3. Reaction conditions were as follows: denaturation at 95°C for 30 seconds, extension for 40 cycles (denaturation at 95°C for 5 seconds, extension at 60°C for 30 seconds) and final extension at 75°C for 5 seconds. Two replicate wells were set for target genes and internal control to calculate the average Ct value. ∆Ct = Ct of target gene − Ct of internal control. 2‐∆Ct was the expression level of LINC00659 relative to GAPDH in gastric cancer tissues. ∆∆Ct = ∆Ct of tumour tissue‐∆Ct of adjacent tissues, and the fold change was 2^−∆∆Ct^, which was the expression in gastric cancer tissues relative to adjacent tissues.

### 2.3 Cell transfection assay

Cell transfection was performed using the Lipofectamine RNAiMAX kit (Invitrogen). siRNA oligonucleotide si‐LINC00659 and si‐negative control of LINC00659 (as control) were transfected (Genepharma synthesis). The siRNA sequence of LINC00659 was Sense: 5'‐CCGGTCCCTCCTTGTGCTTCA‐3'.

Gastric cancer cells in logarithmic phase were digested and adjusted to cell concentration of 10^4^/mL. After cell confluency reached 80%, cell transfection was performed. In brief, 1.5 μL Lipofectamine RNAiMAX solution was diluted with 25 μL of serum‐free Opti‐MEM medium (Gibco). Simultaneously, siRNA (0.5 μL/5 pmol/L) was diluted with 25 of serum‐free Opti‐MEM medium. Afterwards, the diluted siRNA and Lipofectamine RNAiMAX solution were mixed in a 1:1 volume ratio, incubated at room temperature for 5 minutes. Finally, the siRNA complex was added into cells and incubated at 37°C for 48 hours, followed by detection of the expression of LINC00659.

### 2.4 Cell viability by CCK‐8 assay

SGC‐7901 and BGC‐823 cells were divided into Control and si‐LINC00659 groups and inoculated in 96‐well plates. Three replicate wells were set, and blank medium was used as control. Cell viability was measured at 0, 3, 6, 12, 24, 36, 48 hours after transfection. In brief, 100 μL of medium/per well was replaced, followed by addition of 10 μL of CCK‐8 reagent (Beyondtime Biotechnology Co., Ltd.). After incubation for additional 2 hours, the absorbance was measured at 450 nm.

### 2.5 Detection of cell invasion capacity by Transwell chamber

SGC‐7901 and BGC‐823 cells were transfected with siRNA. Matrigel (BD) was melted overnight at 4°C for 24 hours. Afterwards, 300 μL of serum‐free medium was added to 60 μL of Matrigel, mixed and placed in the upper chamber, incubated at 37°C for 4‐5 hours until Matrigel was solid. Cells were suspended in serum‐free DMEM medium, and cell concentration was adjusted, followed by inoculation of cells into the upper chamber. And 500 μL of complete medium containing 20% FBS was placed in the lower chamber. After incubation for 24 hours, the Transwell chamber was taken out, washed with PBS 2 times, fixed with neutral formaldehyde and stained with 0.1% crystal violet solution. Finally, the number of invading cells was observed and counted under microscope.

### 2.6 Cell cycle detection

Cell cycle was detected using PI staining kit (BD) by FACS calibur. Briefly, after siRNA transfection, SGC‐7901 and BGC‐823 cells were cultured for 24 hours, harvested with 0.25% trypsin and 0.02% EDTA, centrifuged at 1000 r/min to collect cells. The resuspended cells were washed with pre‐cooled PBS, fixed in 70% ethanol at 4°C overnight. RNase was added at a final concentration of 50 μg/mL, and PI staining solution was added at 60 μg/mL after water bath. Cell cycle was detected by flow cytometry after incubation in dark for 30 minutes.

### 2.7 Detection of cell migration ability by wound healing assay

After siRNA transfection, SGC‐7901 and BGC‐823 cells were inoculated into 6‐well plates. After cell confluency reached about 70%, s sterile pipette was used for scratching, and sterile cotton swab was utilized to wipe excess cells. Afterwards, culture medium was added for incubation, followed by observation of cell migration degree. Image Pro‐Plus 6.0 software was used for quantitative analysis of migration rate.

### 2.8 Colony formation assay

Colony formation assay was performed to assess the colony formation capacity. In brief, transfected cells were seeded in a 6‐well plate (200‐300 cells/per well) and incubated. Culture medium was changed every 2‐3 days, and colony was formed for about two weeks. After discarding the medium, colonies were fixed with ethanol, stained with 1% crystal violet for 10 minutes. After rinsing with PBS, the number of colony formation was observed and quantitatively analysed.

### 2.9 Detection of protein expression by Western blot

After siRNA transfection, cells were cultured for additional 24 hours. Then, cells were collected, washed twice with PBS and added with RIPA lysate (Beyondtime Biotechnology Co., Ltd.) and PMSF solution (Beyondtime Biotechnology Co., Ltd.) at a ratio of 100:1 for lysis on ice for 0.5 hour. After centrifugation at 3000 *g* for 5 minutes, the precipitate was removed and the supernatant was collected for protein quantification by BCA kit (Beyondtime Biotechnology Co., Ltd.). Protein sample was mixed with 5x loading buffer, boiled for 8 minutes, subjected to 8%‐12% SDS‐PAGE (according to molecular weight) at 80 V and then 120 V and transferred to PVDF membrane at 300 mA for 0.5‐2 hours. The PVDF membranes were blocked with 5% skim milk for 2 hours and incubated with primary antibodies in TBST. The primary antibodies included cell cycle‐regulated proteins p21, Cyclin E, Cyclin D and CDK2 (Abcam, dilution 1:300), PI3K‐AKT‐related proteins PI3K, AKT, p‐AKT and mTOR (Abcam, dilution 1:250). Afterwards, the membranes were washed with TBST twice, incubated with horseradish peroxidase‐labelled goat anti‐rabbit secondary antibody (Abcam, dilution 1:2000). After band visualization with ECL, Image Pro‐Plus 6.0 software was used to analyse optical density. GAPDH was used as the internal control, and results were shown as comparison of the optical density between target protein and internal control.

### 2.10 RNA pull‐down assay

Biotin‐labelled RNAs were transcribed using Biotin RNA Labeling Mix (Roche) and T7 polymerase (Promega), treated by RNAse‐free Dnase I (Promega) and Rneasy Mini Kit (QIAGEN). Then, 3 μg of biotinylated RNA was reacted at 90°C for 2 minutes, then placed on ice for 2 minutes, followed by addition of RNA structure buffer (10 nmol/L Trist pH 7.0, 0.1 mol/L KCl, 10 mmol/L MgCl_2_) and incubation at room temperature for 20 minutes. SGC‐7901 cells were suspended in 2 mL of PBS, added with nuclear isolation buffer, 6 mL of ddH_2_O for incubation at ice for 20 minutes. After centrifugation at 625 *g* for 15 minutes, 1 mL of RIP buffer was suspended in the nucleus, and nucleus was resuspended by homogenizer for 15 times, followed by centrifugation at 325 *g* for 10 minutes. After incubation for 1 hour at room temperature, 20 μL of streptavidin agarose beads was added to each binding reaction and incubated for 30 minutes at room temperature. The agarose beads were washed on the Handee spin column for 5 minutes, to obtain protein after boiling in SDS buffer for Western blot analysis.

### 2.11 Luciferase assay

SGC‐7901 cells were seeded in 24‐well plates at a density of 30%‐40%. The reaction element of SUZ12 (0.1 μg/mL) was added (2.5 μL/well), and DNA: Lipo at a ratio of 1:3 was also added. 50 μL of serum‐free medium was used to dissolve the SUZ12 reaction element and Lipo for 5 minutes. Afterwards, the SUZ12 reaction element and Lipo medium were mixed at a ratio of 1:1, incubated for 20 minutes. The mixture was added to the culture plate (100 μL/per well) and incubated at 37°C for 24 hours. Subsequently, 100 μL of 1 × lysis buffer was added to each well and frozen at 80°C for 30 minutes. Finally, 30 μL sample was transferred into the EP tube, added with 50 μL of Luciferase buffer, mixture, mixed and assessed.

### 2.12 The construction and detection method of mouse model

The animals were male nude mice of the BALB/C‐nu/nu strain (Saiye Biotechnology Co., Ltd.). The mice were 4‐5 weeks old and weighed 19‐23 g. They were raised under SPF‐level ultra‐clean laminar flow. After SGC‐7901 and SGC‐7901‐LINC00659^−/−^cells were cultured to logarithmic phase, the cells were digested and the cell concentration was adjusted to 3 × 10^7^/mL. Nude mice wiped the back of the right hind limb with iodophor in a clean environment and injected the cell suspension, 0.2 mL, the cell volume is 6 × 10^6^. Nodules appeared at the injection site of mice within 3‐4 days. SGC‐7901 and SGC‐7901‐LINC00659^−/−^ cells were inoculated with 10 each. After 14 days, 8 mice were successfully inoculated with SGC‐7901 and 6 mice were successfully inoculated with SGC‐7901‐LINC00659^−/−^. The long diameter of the tumour is 0.3‐1.4 cm. The mice were kept for another 14 days. After 28 days, the mice were killed by carbon dioxide asphyxiation method, and tumour tissues were taken to detect the changes in tumour volume. Western blot method detects the protein levels of SUZ12, p21, Cyclin E, Cyclin D and CDK2.

### 2.13 Statistical analysis

SPSS 21.0 software was used for statistical analysis. Chi‐square test and Fisher's exact probability test were used for clinical physiological and pathological data. Kaplan‐Meier method was utilized to plot survival curve, along with Log‐rank test for difference comparison. Univariate and multivariate Cox hazard regression models were used to investigate the prognostic factors associated with survival rate. Measurement data were shown as mean ± standard deviation ($\bar{\chi }$ ± s). One‐way ANOVA was used for comparison among multiple groups, and SNK test was used for comparison between groups. A *P* < .05 was considered as statistical significance.

## 3 RESULTS

### 3.1 The expression of LINC00659 in gastric cancer and the correlation with clinicopathological characteristics and prognosis

**FIGURE 1:**
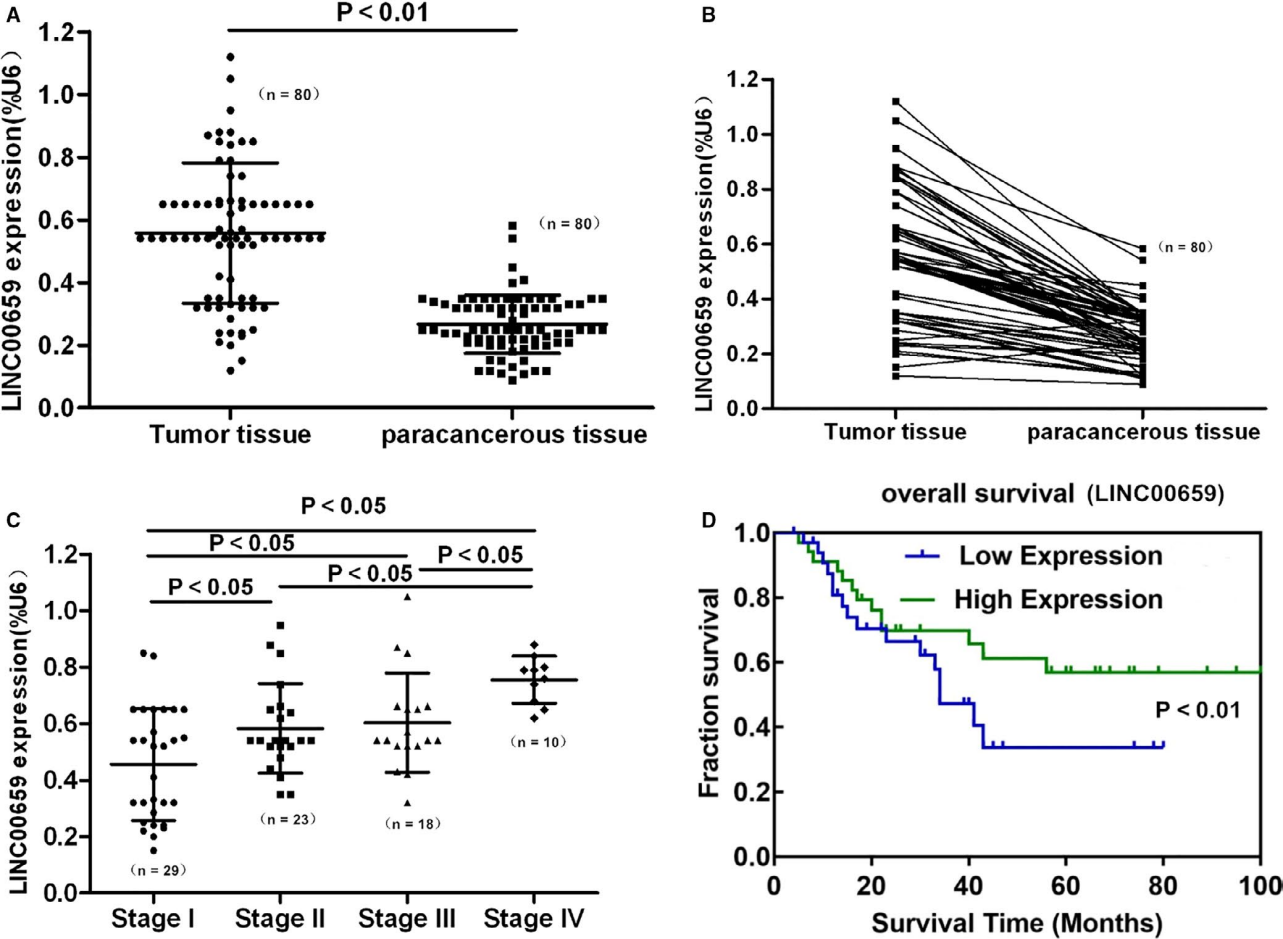
The expression of LINC00659 in gastric cancer and the correlation with clinicopathological characteristics and prognosis. A, Expression level of LINC00659 in tumour tissues and adjacent tissues (n = 80). B, Expression comparison of LINC00659 in tumour tissues and adjacent tissues (n = 80). C, The correlation of LINC00659 expression level in tumour with tumour stage (Stage I: n = 29; Stage II: n = 23; Stage III: n = 18; Stage I: n = 10). D, The correlation of LINC00659 expression with patient survival (Low Expression: n = 40; High Expression: n = 40)

The relative expression of LINC00659 in gastric cancer tissues was (0.53 ± 0.10), which was significantly higher than that in adjacent tissues (0.28 ± 0.08) (*P* < .01). In different tumour stages, LINC00659 was up‐regulated with increasing tumour staging, indicating that LINC00659 was associated with tumour stage (shown in Figure 1A‐C). A total of 80 patients with gastric cancer were divided into LINC00659 high expression group and low expression group (N = 40 each) according to the cut‐off value of 0.58 of LINC00659 relative expression. The correlation analysis between LINC00659 expression and clinicopathological characteristics of gastric cancer patients showed that the expression level of LINC00659 was associated with tumour stage and lymph node metastasis, whereas was not associated with age, gender and histological differentiation. Multivariate regression analysis revealed that lymph node metastasis, tumour stage and LINC00659 expression level were independent prognostic predictors for patients with gastric cancer (shown in Tables 1 and 2). Survival curve analysis demonstrated that patients with low expression of LINC00659 had higher overall survival rate (shown in Figure 1D).

**TABLE 1:** The correlation of LINC00659 in gastric cancer tissue and clinicopathological factors (X^2^ test)

Clinicopathological factors	Number of cases	LINC00659 expression		*P* value
		Low expression (%)	High expression (%)	
Age				
≤55	42	20 (47.6)	22 (52.4)	.185
>55	38	20 (52.6)	18 (47.4)	
Gender				
Male	48	25 (52.1)	23 (47.9)	.251
Female	32	15 (46.9)	17 (53.1)	
Lymph node metastasis				
Positive	48	12 (25.0)	36 (75.0)	.000
Negative	32	28 (87.5)	4 (12.5)	
Differentiation degree				
Well/moderately differentiated	45	23 (51.1)	22 (48.9)	.721
Poorly differentiated	35	17 (45.6)	18 (54.4)	
Local invasion				
T1, T2	50	25 (50.0)	25 (50.0)	.952
T3, T4	30	15 (50.0)	15 (50.0)	
TNM stage				
I‐II stage	52	18 (34.6)	34 (65.4)	.000
III‐IV stage	28	22 (78.6)	6 (21.4)	

**TABLE 2:** Multivariate analysis of prognosis in patients with gastric cancer (n = 80)

Variables	HR	95% CI	*P* value
Lymph node metastasis	2.635	1.325‐6.024	.000
TNM stage	2.754	1.325‐5.241	.001
LINC00659	3.254	1.324‐3.542	.000

### 3.2 The expression of LINC00659 in gastric cancer cells, determination of knock‐down efficiency and effects on cell viability

**FIGURE 2:**
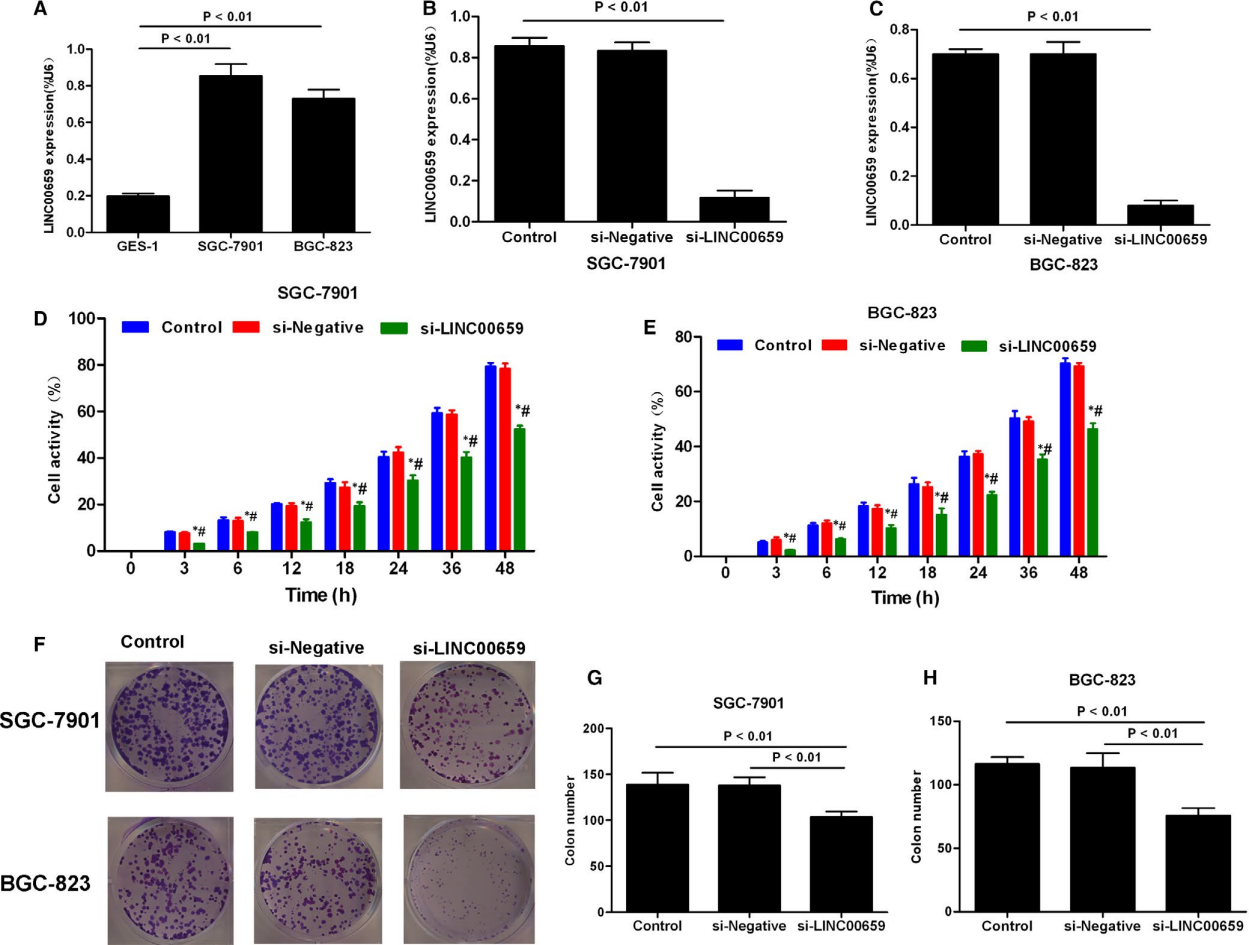
Expression of LINC00659 in gastric cancer cells and its association with cell viability. A, The expression of LINC00659 in gastric cancer cell lines (SGC‐1901 and BGC‐823) and normal cell line, GES‐1. The expression level of LINC00659 was significantly higher in SGC‐7901 and BGC‐823 than that of GES‐1, *P* < .01. B‐C, The determination of siRNA silencing efficiency of LINC00659 in SGC‐7901 and BGC‐823 showed that the expression of LINC00659 in the si‐LINC00659 group was significantly lower than that of Control and si‐Negative (*P* < .01). D‐E, Cell viability by CCK‐8 assay showed that the cell viability was increased within 3‐48 h, which was significantly lower in the si‐LINC00659 group than that of the Control group and the si‐Negative group. Compared with Control group at the same time point, *P < .05; comparison with si‐Negative group, #*P* < .05. F‐H, Colony formation assay showed that the number of colony formed was significantly lower in the si‐LINC00659 group than that in the Control and si‐Negative groups (*P* < .01)

The expression of LINC00659 was significantly higher in gastric cancer cell lines (SGC‐7901 and BGC‐823), compared with that in normal cells (GES‐1) (*P* < .01). After siRNA silencing targeting LINC00659, the expression level of LINC00659 was significantly down‐regulated in SGC‐7901 and BGC‐823 than that in Control group (*P* < .01). Colony formation assay showed that colony formation capacity was significantly weakened than Control group after LINC00659 silencing (*P* < .01). Cell viability assay by CCK‐8 revealed that the cell viability was significantly down‐regulated within 3‐48 hours after LINC00659 silencing, compared with that in Control group (*P* < .01) (shown in Figure 2).

### 3.3 The effects of LINC00659 on cell cycle, migration and invasion ability of gastric cancer cells

**FIGURE 3:**
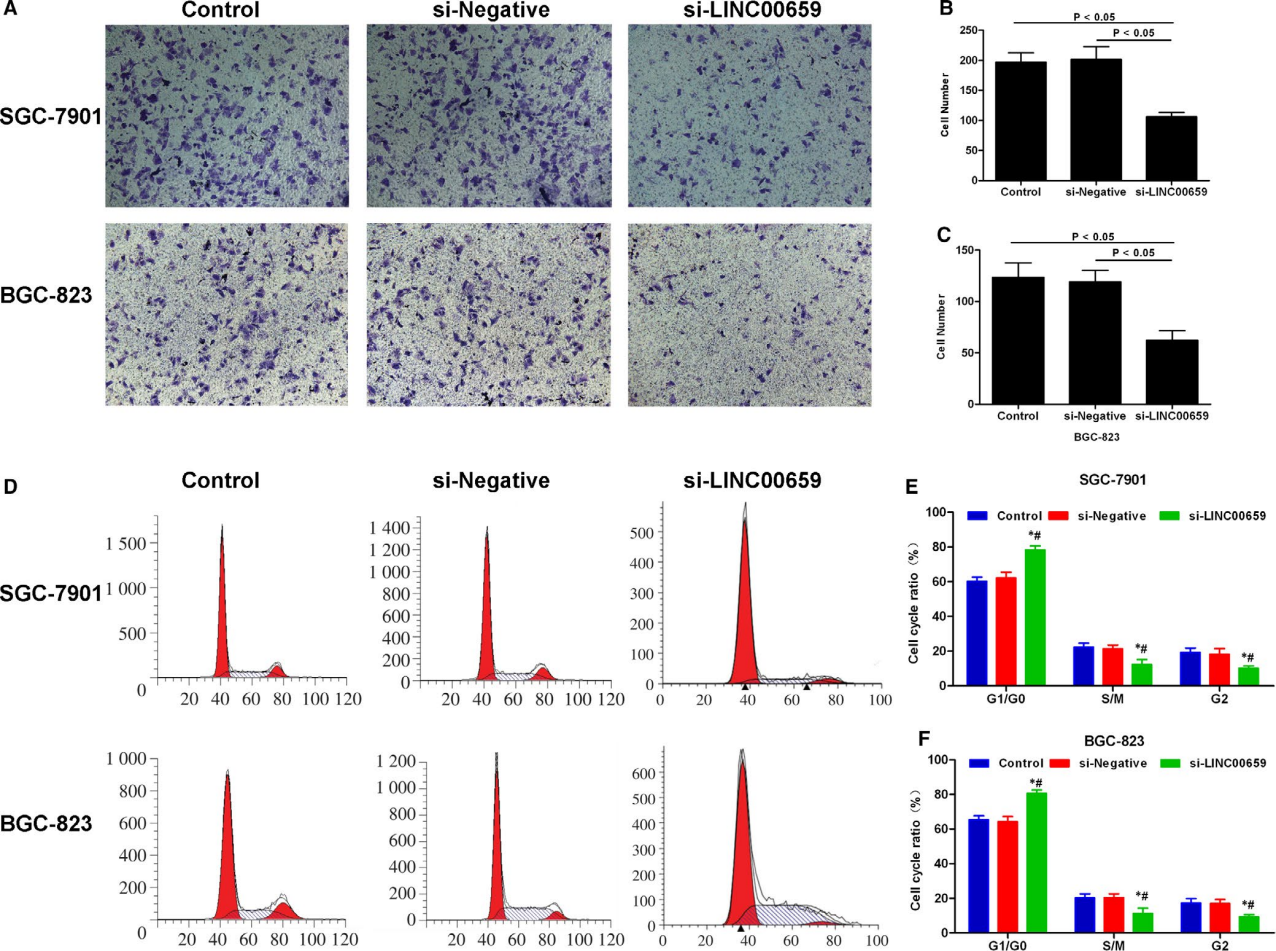
The effects of LINC00659 on cell invasion and cell cycle of gastric cancer. A‐C, The effects of LINC00659 on cell invasion ability. The number of invaded cell was significantly lower in si‐LINC00695 group than that in Control group and si‐Negative group, *P* < .05. D‐F, The effects of LINC00659 on cell cycle. The proportion of G1/G0 was significantly increased, whereas the proportion of S/M phase and G2 phase were significantly decreased in the si‐LINC00695 group. Comparison with Control group *P < .05; Comparison with the si‐Negative group, #*P* < .05

**FIGURE 4:**
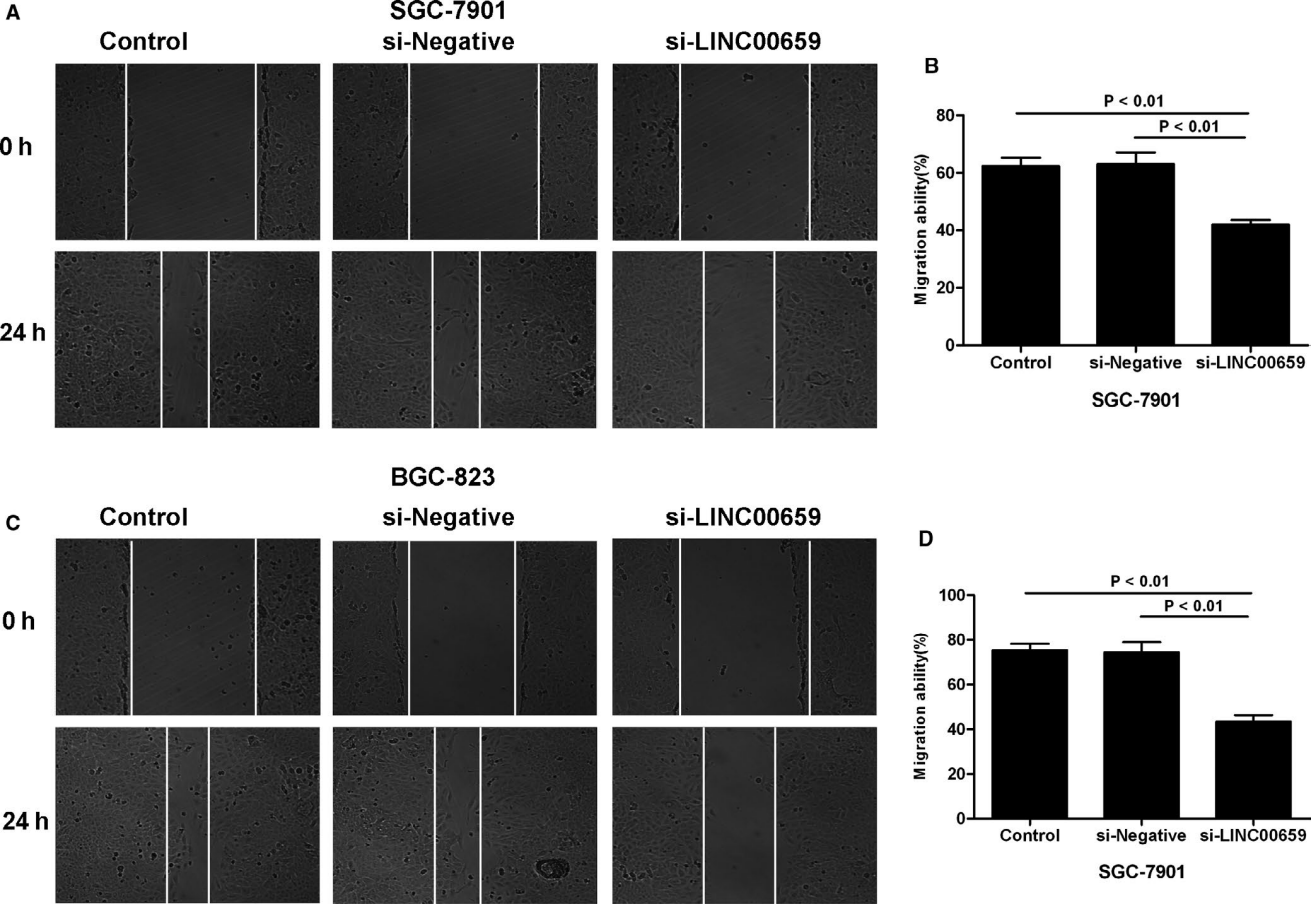
The effects of LINC00695 on cell migration ability. A‐B, The effects of LINC00695 on the migration ability of SGC‐7901 cells. The cell migration ability was significantly down‐regulated in si‐LINC00659 group, in comparison with Control group and si‐Negative group, *P* < .05. C‐D, The effects of LINC00695 on the migration ability of BGC‐823 cells. The cell migration ability was significantly decreased in si‐LINC00659 group, compared with Control group and si‐Negative group, *P* < .05

Transwell assay showed that the invasion ability was significantly down‐regulated and the number of migrated cells was significantly decreased in si‐LINC00659 group, in comparison with those in Control group and si‐Negative group (*P* < .05). Cell cycle analysis showed that the proportion of G1/G0 was significantly increased, whereas the proportions of S/M phase and G2 phase were significantly down‐regulated in si‐LINC00695 group, in comparison with the Control group and the si‐Negative group (*P* < .05). Wound healing assay revealed that the cell migration ability was significantly decreased in the si‐LINC00695 group than that in the Control group and the si‐Negative group (*P* < .05) (shown in Figures 3 and 4).

### 3.4 The regulatory roles of LINC00659 on cell cycle regulating protein and PI3K‐AKT

**FIGURE 5:**
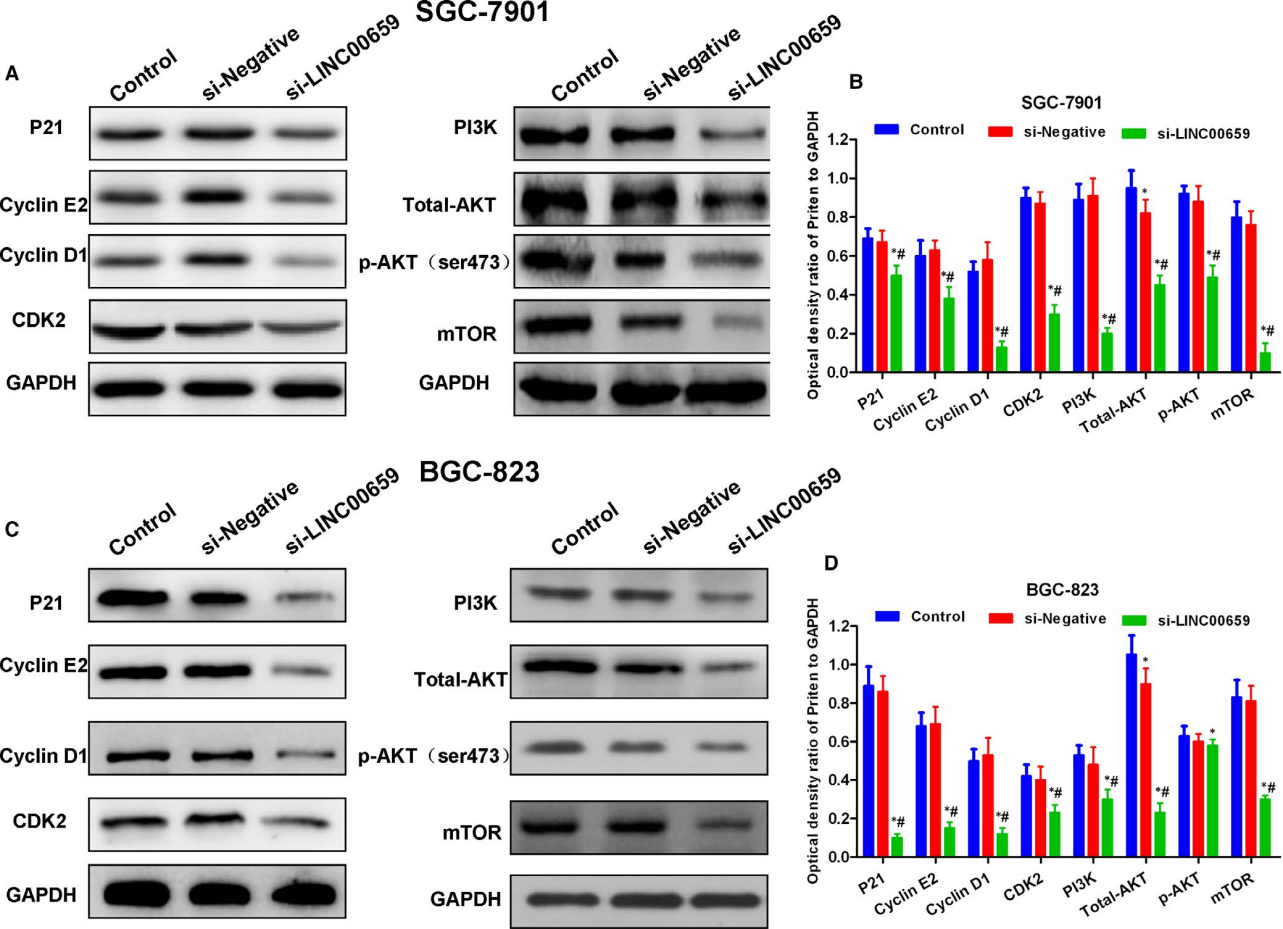
The effects of LINC00659 on cell cycle regulatory protein and PI3K‐AKT signals. A‐B, The effects of LINC00659 on cell cycle regulatory protein and PI3K‐AKT signals in SGC‐7901. The expression of the cell cycle regulatory protein, p21, Cyclin E, Cyclin D and CDK2 was significantly down‐regulated, and PI3K‐AKT signal was suppressed in the si‐LINC00659 group. Comparison with the Control group, *P < .05; comparison with si‐Negative group, #*P* < .05. C‐D, The effects of LINC00659 on cell cycle regulatory protein and PI3K‐AKT signals in BGC‐823. The expression of the cell cycle regulatory protein, p21, Cyclin E, Cyclin D and CDK2 was significantly down‐regulated, and PI3K‐AKT signal was suppressed in the si‐LINC00659 group. Comparison with the Control group, *P < .05; comparison with si‐Negative group, #*P* < .05

In SGC‐7901 and BGC‐823 cells, the expression of key proteins in regulating cell cycle, p21, Cyclin E, Cyclin D and CDK2 was significantly down‐regulated in the si‐LINC00659 group than that in the Control and si‐Negative groups (*P* < .05). Meanwhile, the PI3K‐AKT signal was inhibited. The expression of key protein, PI3K, AKT, p‐AKT and mTOR was significantly suppressed in the si‐LINC00659 group, in comparison with Control group and si‐Negative group (*P* < .05) (shown in Figure 5).

### 3.5 Targeted regulation of transcription factor SUZ12 by LINC00659

In this study, we used co‐expression analysis to find that LINC00659 and SUZ12 have regulatory relationship. At the same time, LINC00659 also has a variety of regulatory relationships. As a transcription factor, SUZ12 can transcribe a variety of proteins to regulate the biological behaviour of tumours, so this study further verified by RNA pull‐down and other experiments.

**FIGURE 6:**
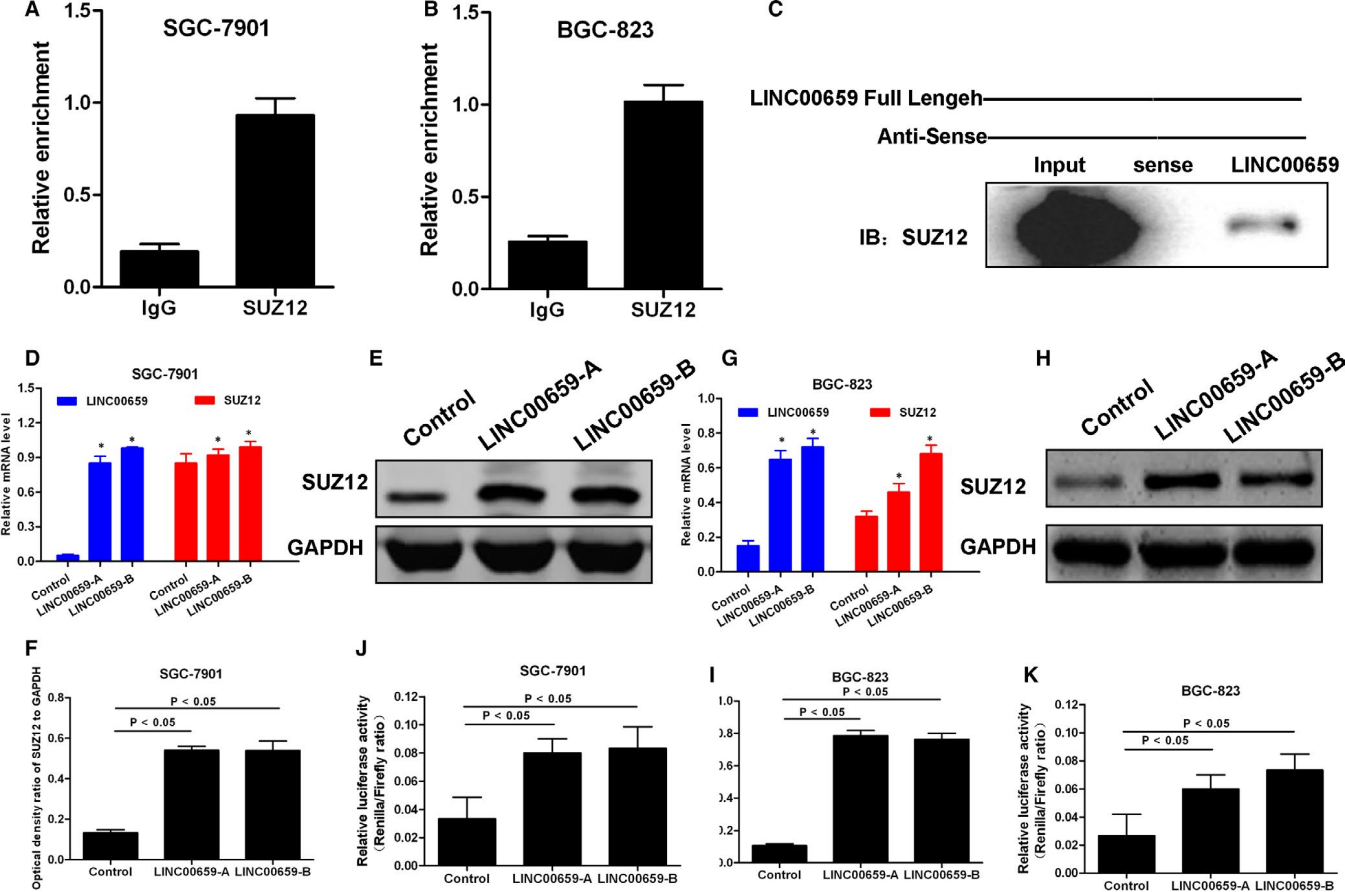
The targeted regulation relationship between LINC00659 and SUZ12. A‐B, RNA‐IP assay confirmed that LINC00659 could bind with SUZ12 in gastric cancer cells, indicating the targeted relationship between the two. C, RNA pull‐down assay validated the targeted relationship between LINC00659 and SUZ12. The expression of SUZ12 was increased after pulling down the protein, confirming the binding relationship between the two. D‐F, The overexpression of LINC00659 caused increased expression level of both LINC00659 and SUZ12 in SGC‐7901 cells. G‐I, The overexpression of LINC00659 caused increased expression level of both LINC00659 and SUZ12 in BGC‐823cells. J‐K, Luciferase reporter gene assay showed the targeted relationship between LINC00659 and SUZ12

Co‐expression analysis showed that transcription factors SUZ12 were correlated with LINC00659. RNA‐IP and RNA pull‐down assays further showed that LINC00659 could bind to SUZ12 in SGC‐7901 and BGC‐823 cells. After transfection with LINC00659 overexpressing lentiviral vector (LINC00659‐A and LINC00659‐B), Western blot analysis showed that the expression of SUZ12 was significantly increased in SGC‐7901 and BGC‐823, and luciferase reporter assay revealed that the activity of SUZ12 was significantly enhanced after overexpression of LINC00659 in SGC‐7901 and BGC‐823. Therefore, LINC00659 could regulate the expression of transcription factor SUZ12 (shown in Figure 6).

### 3.6 SUZ12 rescue assay verified the action mechanism of LINC00659 in SGC‐7901

**FIGURE 7:**
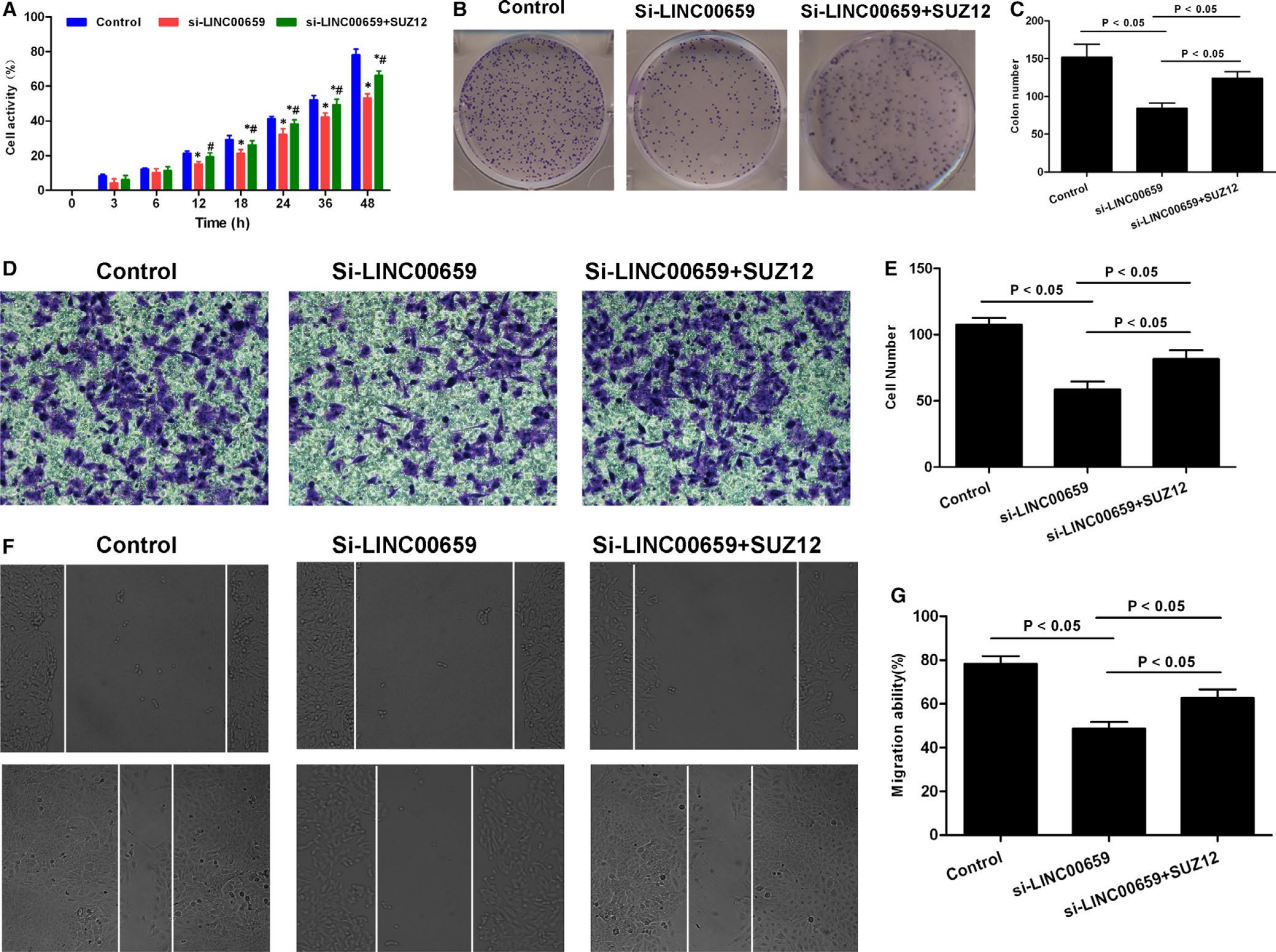
SUZ12 rescue assay on cell viability, migration and invasion ability of SGC‐7901 cells. A, Cell viability by CCK‐8 assay showed that cell viability was significantly decreased in si‐LINC‐00659 group than that in Control, whereas cell viability was significantly enhanced in si‐LINC‐00659 + SUZ12 group compared with that in si‐LINC‐00659 group. Compared with Control group, *P < .05; Comparison with si‐LINC00659 group, #*P* < .05. B‐C, Colony formation assay revealed that the number of colony formed was significantly lower in si‐LINC00659 than that of Control group, whereas the number of colony formed was significantly higher in si‐LINC‐00659 + SUZ12 than that of si‐LINC00659 group. Comparison between groups, *P* < .05. D‐E, Transwell assay showed that the number of invaded cells was lower in si‐LINC00659 group than that of Control group, whereas the number of invaded cells was significantly higher in si‐LINC‐00659 + SUZ12 group than that of si‐LINC00659 group. Comparison between groups, *P* < .05. F‐G, Wound healing assay showed lower migration ability in si‐LINC00659 than that in control group, whereas cell migration ability was significantly higher in si‐LINC‐00659 + SUZ12 group than that si‐LINC00659 group. Comparison between groups, *P* < .05

**FIGURE 8:**
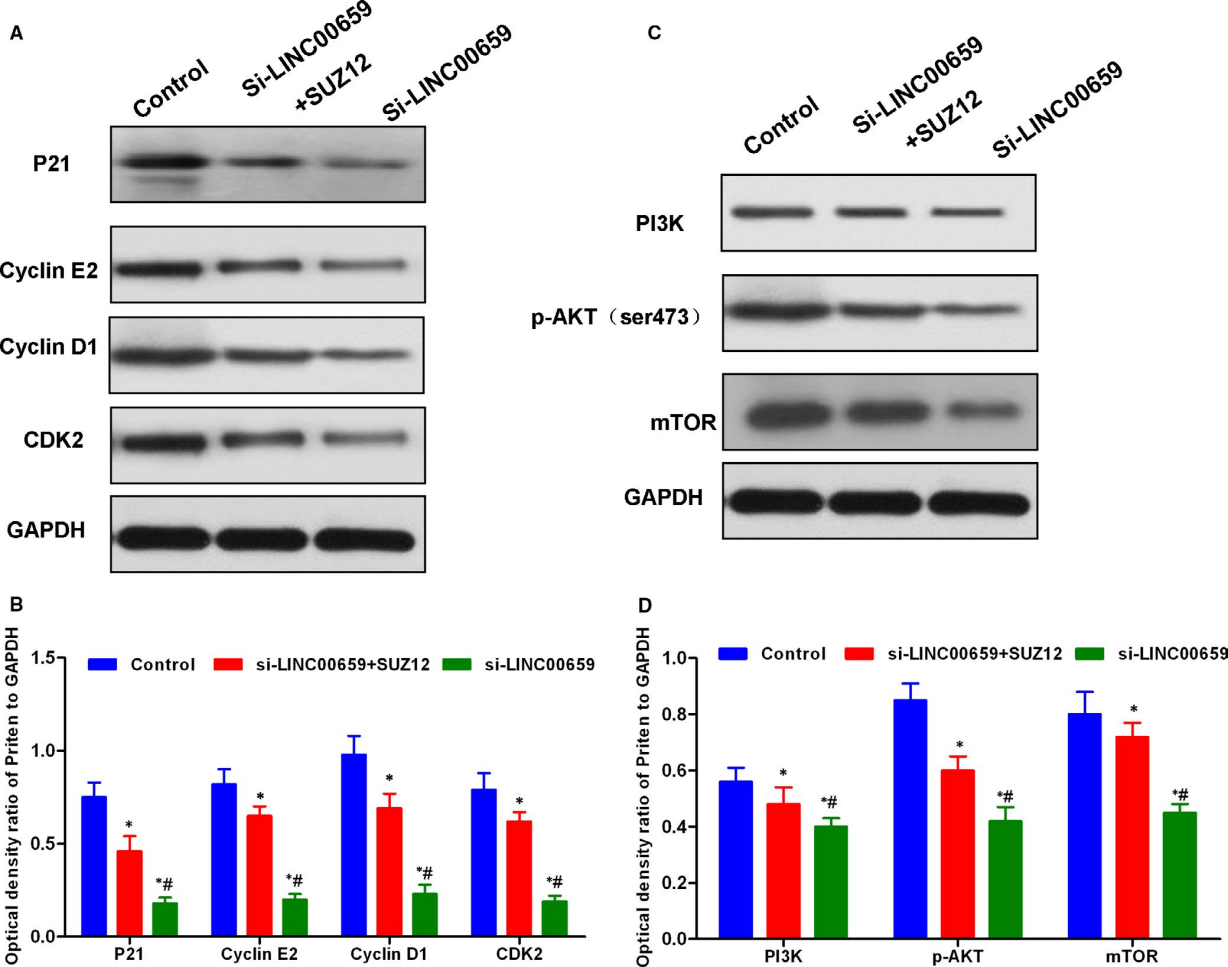
SUZ12 rescue assays on cell cycle regulatory proteins and PI3K‐AKT signalling. A‐B, The expression level of key proteins of cell cycle. The relative expression levels of the cell cycle regulatory proteins P21, Cyclin E2, Cyclin D1 and CDK2 were significantly lower in the si‐LINC00659 group than those in the Control group and the si‐LINC00659 + SUZ12 group. After SUZ12 rescue, the level of cell cycle regulatory protein was increased. Comparison with the Control group, *P < .05; comparison with si‐LINC00659 + SUZ12 group, #*P* < .05. B‐D, The expression level of key proteins in PI3K‐AKT signal. The relative expression levels of PI3K, p‐AKT and mTOR were significantly lower in si‐LINC00659 group than those in Control group and si‐LINC00659 + SUZ12 group. After SUZ12 rescue, the level of PI3K‐AKT signalling protein was increased. Comparison with the Control group, *P < .05; Comparison with the si‐LINC00659 + SUZ12 group, #*P* < .05

To further investigate that SUZ12 was a target protein of LINC00659, SGC‐7901 cell line was transfected for SUZ12 overexpression. Briefly, SGC‐12 cells were divided into Control, si‐LINC00659, si‐LINC00659 + SUZ12 groups, followed by cell viability assay, colony formation assay, wound healing assay and Transwell chamber assay. In addition, Western blot was used to detect the levels of cell cycle regulatory proteins p21, Cyclin E, Cyclin D, and CDK2 and PI3K‐AKT signal. As a result, SUZ12 overexpression led to significantly increased colony formation ability, migration capacity and invasion ability in si‐LINC00659 + SUZ12 group, compared with those of si‐LINC00659 group (*P* < .05). Meanwhile, the expression cell cycle regulatory protein was significantly higher in si‐LINC00659 + SUZ12 group than that of si‐LINC00659 group. And PI3K signal was activated. These results showed that SUZ12 overexpression could resist the effects caused by down‐regulation of LINC00659 (shown in Figures 7 and 8).

### 3.7 Tumour size comparison and protein expression level

**FIGURE 9:**
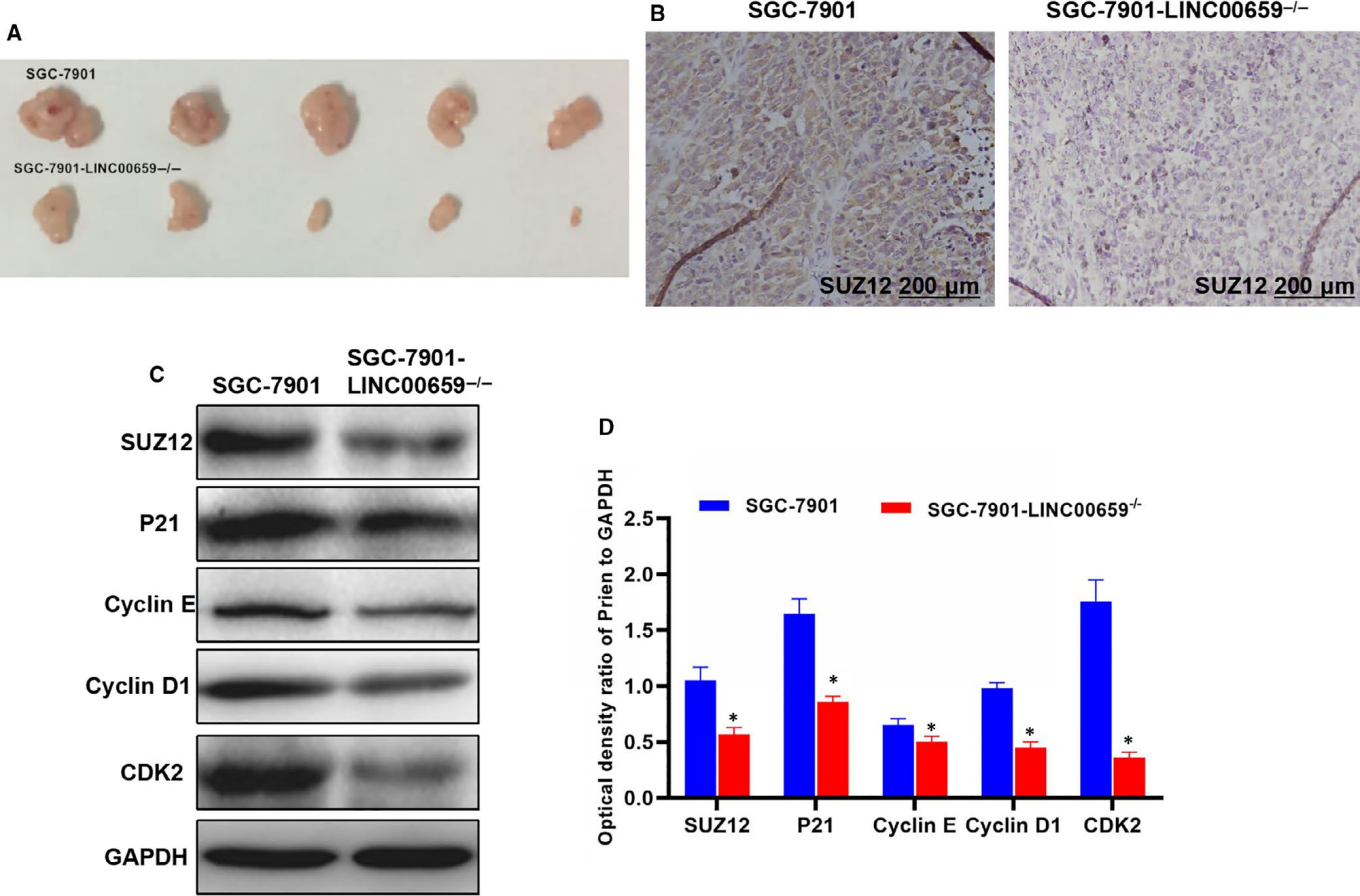
Tumour volume and related protein expression. A, The size of mice in group SGC‐7901 was significantly higher than that in group SGC‐7901‐LINC00659^−/−^. LINC00659 inhibition can significantly inhibit tumour growth. B, The result of immunohistochemical staining method for SUZ12 expression in tumour tissuesThe level of SUZ12 in SGC‐7901 was significantly higher than that in SGC‐7901‐LINC00659^−/−^. LINC00659 inhibition can significantly reduce the expression of SUZ12. C‐D, The expression level of key proteins. The relative expression levels of SUZ12, p21, Cyclin E, Cyclin D and CDK2 were significantly lower in SGC‐7901‐LINC00659^−/−^ than those in SGC‐7901. Comparison with the SGC‐7901, *P < .05

The tumour volume of mice after SGC‐7901 cell inoculation was significantly higher than that of SGC‐7901‐LINC00659^−/−^. After LINC00659 inhibition, the protein levels of SUZ12, p21, Cyclin E, Cyclin D and CDK2 in SGC‐7901 tumour tissue were significantly higher than those of SGC‐7901‐LINC00659^−/−^ (shown in Figure 9).

## 4 DISCUSSION

Targeted therapy and diagnostic markers are new directions in the research field of oncology, and molecular biology and genetic research provide a solid theoretical basis for precision therapy. Substantial progress has been made of LncRNAs in tumours. LncRNAs regulate some classical signalling pathways, such as Notch, mTOR and NF‐κB, and many abnormally expressed LncRNAs are of clinical significance for the diagnosis of gastric cancer. These LncRNAs are not only associated with the metastasis and invasion of gastric cancer, but also closely related to the staging, prognosis and tumour size of gastric cancer. H19, a LncRNA located on chromosome 11p15.5, is abnormally expressed in various tumours. H19 has been revealed to be associated with the proliferation of gastric cancer cells, and H19 can inhibit the apoptosis of gastric cancer.^9^, ^10^ HOTAIR, a non‐coding RNA first discovered in breast cancer, is associated with metastasis and poor prognosis of breast cancer. Interference with HOTAIR can lead to cell cycle arrest at G0/G1 phase of KATOIII, MKN74 and MKN28 and inhibit cell proliferation.^11^, ^12^ In this study, we first validated that the expression of LINC00659 was increased in gastric cancer tissues. Additionally, the expression level of LINC00659 was associated with the stage and lymph node metastasis but was not correlated with age, gender or histological differentiation. Multivariate regression analysis showed that lymph node metastasis, tumour stage and LINC00659 level were independent prognostic predictors for gastric cancer. Survival curves showed that patients with high expression of LINC00659 had shorter OS, whereas patients with lower expression harboured longer survival. Therefore, the preliminary clinical data showed that LINC00659 was associated with the prognosis of gastric cancer.

The expression of LINC00659 was increased in gastric cancer cell lines, SGC‐7901 and BGC‐823. To further study its mechanism of action, the expression of LINC00659 was silenced by siRNA. As a result, the inhibition of LINC00659 resulted in suppressed cell viability, colony formation capacity, migration and invasion ability. The role LINC0069 was similar in two tumour cell lines, indicating the universal effects of this non‐coding RNA in gastric cancer. Co‐expression analysis further revealed the correlation between transcription factors SUZ12 and LINC00659. RNA‐IP and RNA pull‐down assay validated that LINC00659 could bind to SUZ12 in SGC‐7901 and BGC‐823 cells. SUZ12 is a transcription factor and a subunit of the PRC2 complex. Present studied have shown that SUZ12 is associated with tumour proliferation and invasion in lung cancer, gastric cancer, colon cancer, whereas the deletion of SUZ12 gene can significantly inhibit tumour transformation.^13^, ^14^ Moreover, it has been reported that SUZ12 is correlated with the expression of ZNF198 in hepatic carcinoma, and their interaction promotes the proliferation and invasion of hepatic carcinoma.^15^ As a transcription factor, SUZ12 exerts a certain regulatory effect on cyclins. In the study, rescue assay showed that after SUZ12 overexpression, cell proliferation ability was restored, and the key cell cycle regulatory proteins were expressed. CDK, a cyclin‐dependent protein kinase, plays a central role in the cell cycle regulatory network. Among different CDKs, CDK2 is one of the most important ones. CDK2 mainly binds to Cyclin D and Cyclin E and participates in cell differentiation and cell cycle. CDK2 can also regulate the transition from G1 to S phase. In particular, the abnormal expression of CDK2 protein is detected in a variety of tumours, which is related to the proliferation of tumour cells.^16^, ^17^ The expression of Cyclins is significantly periodic and specific, and various signals and transcription factors of intracellular network can regulate the action of Cyclins protein. Cyclin D, a well‐studied protein, is an initial factor of cell cycle. Cells are progressed from G0 phase to G1 phase mediated by Cyclin D through G protein ras and mitotic activation protein kinase (MAPK). Meanwhile, Cyclin D can also bind to CDK4 and CDK6 to shorten the G1 phase. The abnormal expression of Cyclin D in tumour cells can reduce the dependence of cell proliferation on mitogens, causing abnormal cell cycle and abnormal proliferation.^18^, ^19^ PI3K is a key signal in regulating cell proliferation/apoptosis. AKT, the downstream protein of PI3K, can be phosphorylated into p‐AKT to further regulate cyclin.^20^ PI3K can also transmit mitotic signals to p70S6K1 via AKT‐mTOR. And PI3K inhibition can decrease the expression of Cyclin D1, causing G1 phase arrest. In addition, mTOR inhibitors could also lead to similar effects.^21^ Therefore, these studies demonstrate that SUZ12 may play a role by promoting the expression of cyclins and by activating PI3K signals.

## 5 CONCLUSIONS

In summary, here, we show that LINC00659 is involved in the carcinogenesis and progression of gastric cancer. The high expression of LINC00659 can promote the proliferation, metastasis and invasion of gastric cancer, which is related to the promotion of SUZ12 transcription factor expression. Moreover, SUZ12 further plays a role by promoting the expression of cell cycle regulatory proteins.

## CONFLICT OF INTEREST

The authors declare that they have no conflict of interest.

## AUTHOR CONTRIBUTION

**Yongjia Sheng:** Conceptualization (equal); Investigation (equal). **Chenyang Han:** Funding acquisition (equal); Investigation (equal); Methodology (equal). **Yi Yang:** Data curation (equal); Formal analysis (equal); Validation (equal); Visualization (equal); Writing‐original draft (equal). **Jin Wang:** Formal analysis (equal); Supervision (equal); Writing‐review & editing (equal). **Yanling Gu:** Conceptualization (equal); Investigation (equal); Methodology (equal); Visualization (equal). **Wenyan Li:** Conceptualization (equal); Investigation (equal); Methodology (equal); Project administration (equal); Software (equal); Writing‐original draft (equal). **Li Guo:** Methodology (equal); Project administration (equal); Supervision (equal); Writing‐review & editing (equal).

## ETHICAL APPROVAL AND CONSENT TO PARTICIPATE

The study approved with Ethics Committee.

## CONSENT FOR PUBLICATION

All authors approval published the article.

## Data Availability

The data and material were availability.
