# Corrigendum

**DOI:** 10.1111/jcmm.17234

**Published:** 2022-04-05

In Yongjia Sheng et al.,^1^ the published article contains errors in Figure 3A, Figure 5A and Figure 8A. The correct figures are shown below. The authors confirm all results and conclusions of this article remain unchanged.

**FIGURE 3:**
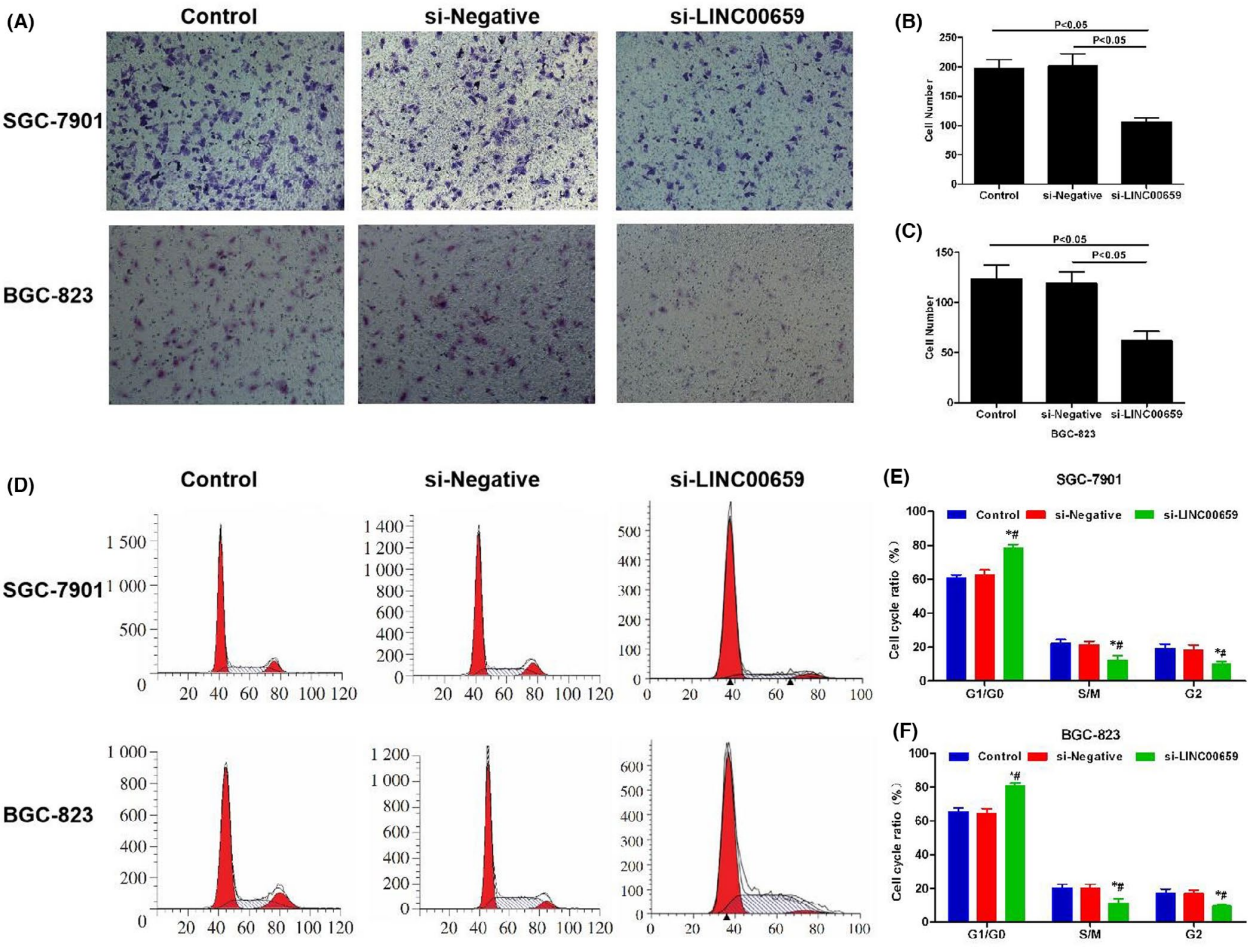
The effects of LINC00659 on cell invasion and cell cycle of gastric cancer. (A–C) The effects of LINC00659 on cell invasion ability. The number of invaded cell was significantly lower in si‐LINC00695 group than that in Control group and si‐Negative group, *p* < 0.05. (D–F) The effects of LINC00659 on cell cycle. The proportion of G1/G0 was significantly increased, whereas the proportion of S/M phase and G2 phase were significantly decreased in the si‐LINC00695 group. Comparison with Control group *p < 0.05; Comparison with the si‐Negative group, ^#^ *p* < 0.05

**FIGURE 5:**
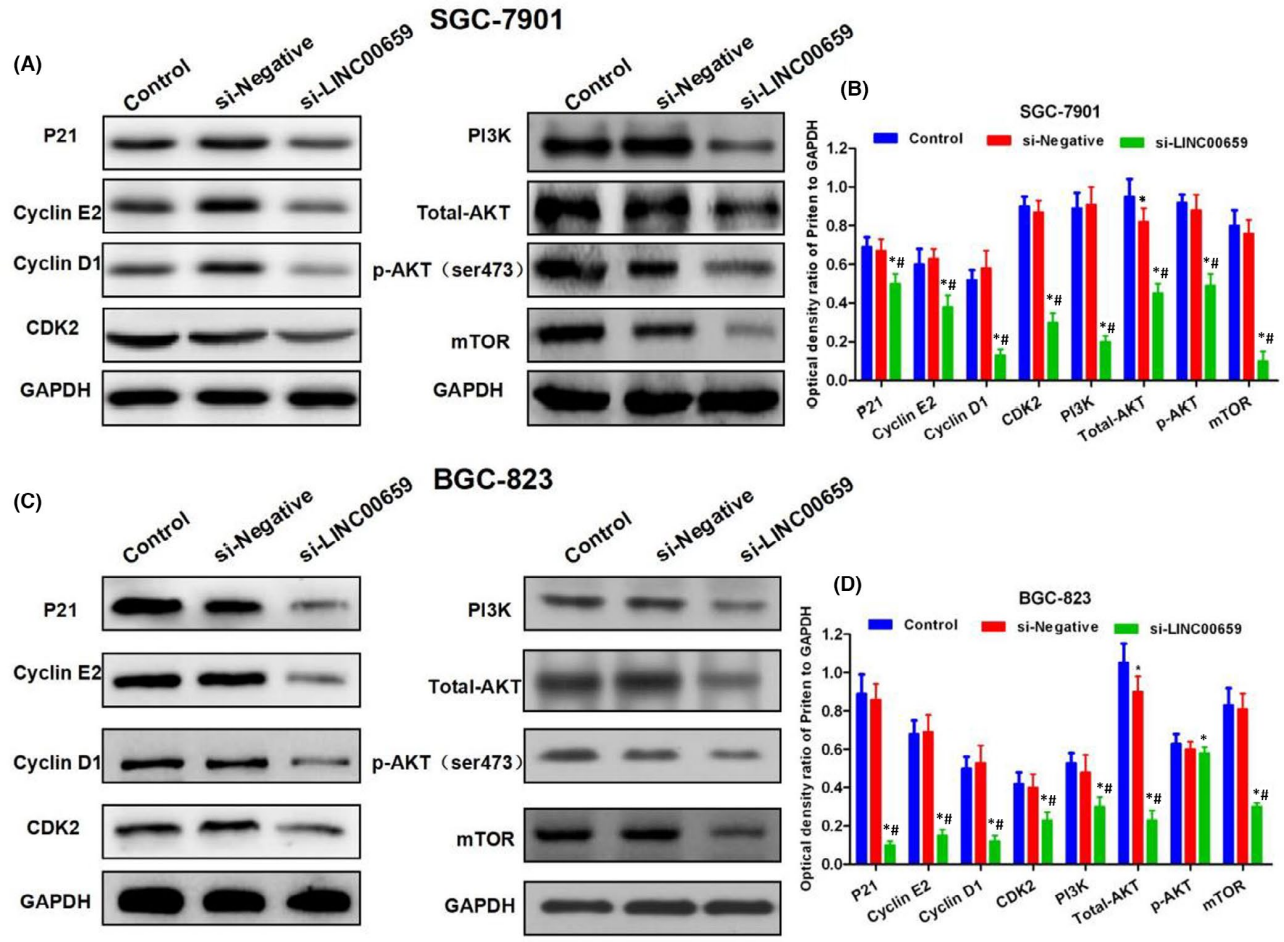
Effects of LINC00659 on cell cycle regulatory protein and PI3K‐AKT signals. (A,B) The effects of LINC00659 on cell cycle regulatory protein and PI3K‐AKT signals in SGC‐7901. The expression of the cell cycle regulatory protein, p21, Cyclin E, Cyclin D and CDK2 was significantly downregulated, and PI3K‐AKT signal was suppressed in the si‐LINC00659 group. Comparison with the Control group, *p < 0.05; comparison with si‐Negative group, ^#^ *p* < 0.05. (C,D) The effects of LINC00659 on cell cycle regulatory protein and PI3K‐AKT signals in BGC‐823. The expression of the cell cycle regulatory protein, p21, Cyclin E, Cyclin D and CDK2 was significantly downregulated, and PI3K‐AKT signal was suppressed in the si‐LINC00659 group. Comparison with the Control group, *p < 0.05; comparison with si‐Negative group, ^#^ *p* < 0.05

**FIGURE 8:**
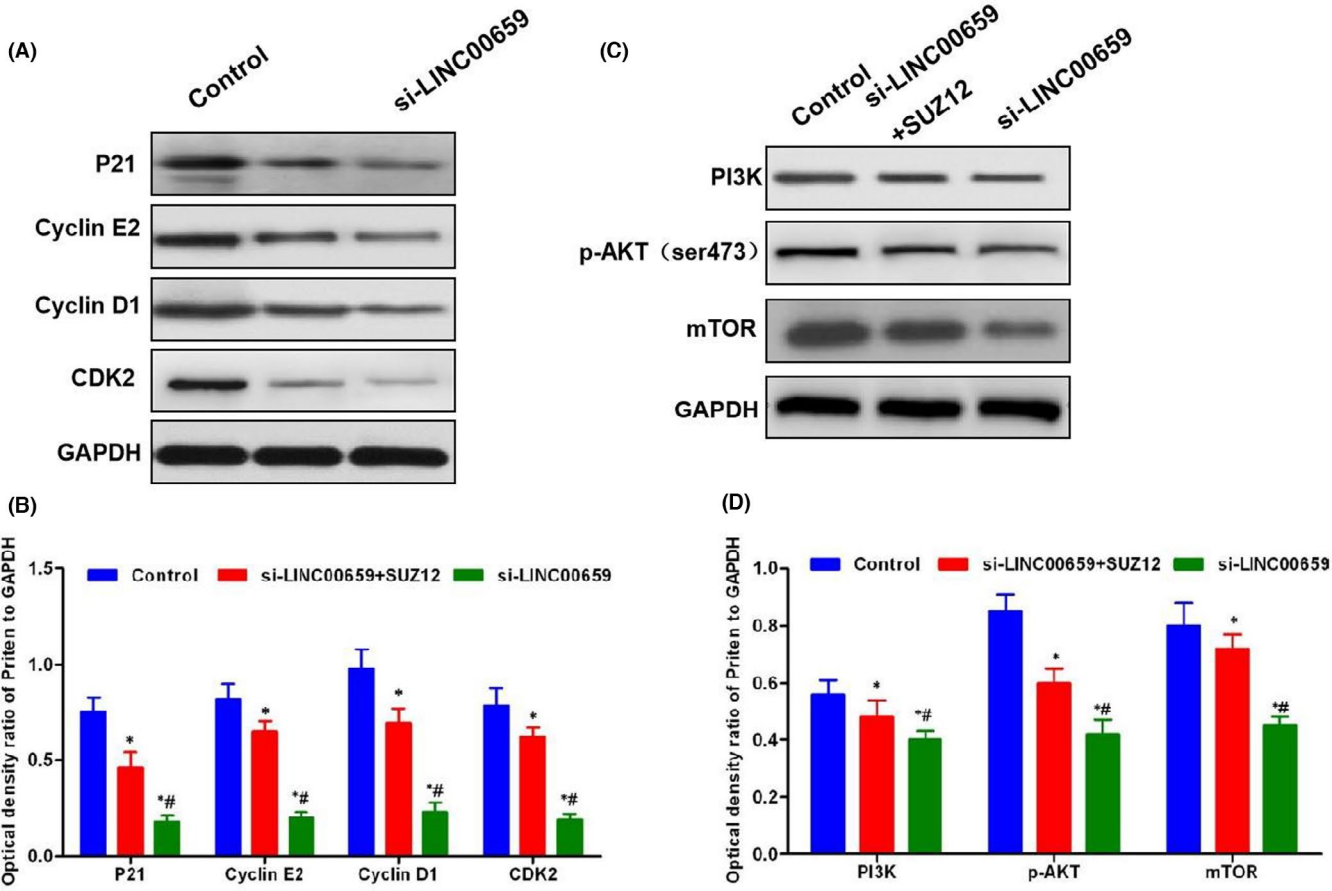
SUZ12 rescue assays on cell cycle regulatory proteins and PI3K‐AKT signalling. (A,B) The expression level of key proteins of cell cycle. The relative expression levels of the cell cycle regulatory proteins P21, Cyclin E2, Cyclin D1 and CDK2 were significantly lower in the si‐LINC00659 group than those in the Control group and the si‐LINC00659 + SUZ12 group. After SUZ12 rescue, the level of cell cycle regulatory protein was increased. Comparison with the Control group, *p < 0.05; comparison with si‐LINC00659 + SUZ12 group, ^#^ *p* < 0.05. (B–D) The expression level of key proteins in PI3K‐AKT signal. The relative expression levels of PI3K, p‐AKT and mTOR were significantly lower in si‐LINC00659 group than those in Control group and si‐LINC00659 + SUZ12 group. After SUZ12 rescue, the level of PI3K‐AKT signalling protein was increased. Comparison with the Control group, *p < 0.05; Comparison with the si‐LINC00659 + SUZ12 group, ^#^ *p* < 0.05
